# The Reduced Folate Carrier (*RFC-1*) 80A>G Polymorphism and Maternal Risk of Having a Child with Down Syndrome: A Meta-Analysis

**DOI:** 10.3390/nu5072551

**Published:** 2013-07-05

**Authors:** Fabio Coppedè, Valentina Lorenzoni, Lucia Migliore

**Affiliations:** 1 Department of Translational Research and New Technologies in Medicine and Surgery, Division of Medical Genetics, University of Pisa, Via S. Giuseppe 22, Pisa 56126, Italy; E-Mail: l.migliore@geog.unipi.it; 2 Institute of Management, Sant’Anna School of Advanced Studies, Pisa 56126, Italy; E-Mail: v.lorenzoni@sssup.it

**Keywords:** reduced folate carrier, SLC19A1, RFC-1, RFC1 A80G polymorphism, RFC1 80A>G polymorphism, Down Syndrome, maternal risk, meta-analysis, folate, gene polymorphisms

## Abstract

A common polymorphism (c.80A>G) in the gene coding for the reduced folate carrier (*SLC19A1*, commonly known as *RFC-1*) has been associated with maternal risk of the birth of a child with Down Syndrome (DS), but results are controversial. We searched major online databases to identify available case-control studies, and performed a meta-analysis to summarize the data concerning this association. Nine independent case-control studies were identified for a total of 930 DS mothers (MDS) and 1240 control mothers. Odds ratios (OR) and 95% confidence intervals (CI) were calculated using both fixed and random effects models. An increase in the risk of having a birth with DS was observed for carriers of the homozygous GG genotype (OR 1.27, 95% CI 1.04–1.57; *p* = 0.02, fixed effects model), even after removal from the meta-analysis of published data with deviations from Hardy-Weinberg equilibrium (HWE) in controls (OR 1.26, 95% CI 1.02–1.55; *p* = 0.03, fixed effects model). Moreover, the pooled OR under the fixed effects model showed an increase in the maternal risk for the G allele (OR 1.14, 95% CI 1.01–1.30; *p* = 0.03). Present results suggest that the maternal *RFC-1* 80A>G polymorphism might be associated with an increased risk of having a birth with DS, particularly among carriers of the GG genotype.

## 1. Introduction

Green vegetables, fruits, cereals, calf’s liver and beans are the major sources of dietary folates whose metabolism, also referred to as one-carbon metabolism, is required for the production of the major intracellular methylating agent *S*-adenosylmethionine (SAM), and for the synthesis of DNA and RNA precursors [1]. Folates are highly hydrophilic molecules that do not cross biological membranes by diffusion alone, but use several transport systems to enter the cells. By far the best characterized folate transporter is the ubiquitously expressed reduced folate carrier (RFC-1) that participates in the uptake of folate cofactors from the blood [2]. There is also evidence of a role for RFC-1 in specialized tissue functions such as absorption across the luminal epithelium in intestine, transplacental transport of folates, folate uptake across the blood-brain-barrier, and transport across the basolateral membrane of renal tubules [2]. A deficiency in cellular folates results in aberrant DNA methylation, point mutations, chromosome breakage, increased frequency of micronuclei, as well as in defective chromosome recombination and aneuploidy [3], and has been linked to several human pathologies including cancer, congenital diseases, cardiovascular diseases, neurological and neuropsychiatric disorders, among others [4].

Primary trisomy 21 leading to Down Syndrome (DS) is caused by the failure of normal chromosome 21 segregation during meiosis. In almost 95% of the cases the nondisjunction event is of maternal origin, occurring primarily during meiosis I in the maturing oocyte [5]. Advancing maternal age at conception and the location of genetic recombination represent the two most important risk factors for chromosome 21 nondisjunction so far identified [6,7]. The cellular and molecular mechanisms that underlie meiotic nondisjunction in DS are still largely unknown, but recent studies have demonstrated a link between DNA methylation of pericentromeric regions and meiotic crossover frequency, suggesting that regional epigenetic organization can pattern recombination frequency along eukaryotic chromosomes [8,9,10].

In 1999, James and coworkers observed that impairments of one-carbon metabolism, due to the presence of polymorphic genes, could be maternal risk factors for the birth of a child with DS [11], and subsequent *in vitro* studies revealed that folate deficiency induces chromosome 21 aneuploidy [12,13]. Those papers have stimulated considerable research in the field, and several case-control studies have been performed to investigate the contribution of maternal polymorphisms of genes involved in one-carbon metabolism as risk factors for having a child with DS (reviewed in [14]). Unfortunately, most of those studies have been conducted in small cohorts of less than, or about, 100 case mothers each, and were often underpowered to evaluate the independent contribution of each studied polymorphism to the maternal risk for trisomy 21 in the offspring [14]. Meta-analyses of published data have been performed to overcome the limits of small case-control cohorts, revealing that both the methylenetetrahydrofolate reductase (*MTHFR* c.677C>T) and the methionine synthase reductase (*MTRR* c.66A>G) polymorphisms (both genes are involved in folate metabolism) might represent independent maternal risk factors for the birth of a child with DS [15,16,17]. Other polymorphic genes participating in one-carbon metabolism have been studied less extensively than the two previous ones, and results are still borderline or inconclusive for most of them [15].

In 2000, Chango and coworkers [18] identified a common c.80A>G polymorphism in the gene coding for RFC-1 (*SLC19A1* gene, commonly known as *RFC-1* gene), that was associated with increased plasma homocysteine (hcy) and decreased folate levels in combination with the *MTHFR* 677C>T one [18]. In 2006, we first suggested a contribution of the *RFC-1* 80A>G polymorphism to the maternal risk of birth of a child with DS, observing association with maternal risk in combination with *MTHFR* 677C>T or *MTHFR* 1298A>C polymorphisms [19]. Subsequent studies have been conflicting with some authors observing an independent association of the *RFC-1* 80A>G polymorphism with the maternal risk of DS in the offspring [20,21] or even more complex interactions among *RFC-1* and other polymorphisms in genes involved in one-carbon metabolism [22,23,24], and others failing to find any association of *RFC-1* 80A>G alone or combined [25,26,27]. Moreover, the *RFC-1* 80A>G polymorphism has been associated with reduced red cell folate concentrations among women [28], and with reduced serum folate concentrations in mothers of DS individuals (MDS) [29]. In addition, the *RFC-1* gene maps to chromosome 21, is over-expressed in DS individuals, and might contribute to impaired one carbon metabolism and to the severity of the DS phenotype [30,31]. In this regard, maternal *RFC-1* polymorphisms have been associated with congenital heart disease in the DS child [32].

A meta-analysis [15] of four genetic association studies [19,20,22,25], for a total of 354 MDS and 644 control mothers, was performed in 2009 to address the role of the *RFC-1* 80A>G polymorphism as a maternal risk factor for the birth of a child with DS, and showed a trend toward an association under the dominant model (GG + AG *vs*. AA) with an odds ratio (OR) of 1.32 (95% CI = 0.95–1.82) [15], suggesting the need of additional studies to further address the contribution of this polymorphism to the maternal risk of having a DS child. Since several additional papers have been published in recent years, including studies in Asian [21,23], European [27], and Brazilian populations [24,26,29], we performed the present meta-analysis of the genetic association studies that investigated the *RFC-1* 80A>G polymorphism as a maternal risk factor for having a birth with DS, including nine independent case-control studies (Table 1) for a total of 930 MDS and 1240 control mothers.

## 2. Experimental Section

### 2.1. Selection of Manuscripts for Meta-Analysis

**Figure 1:**
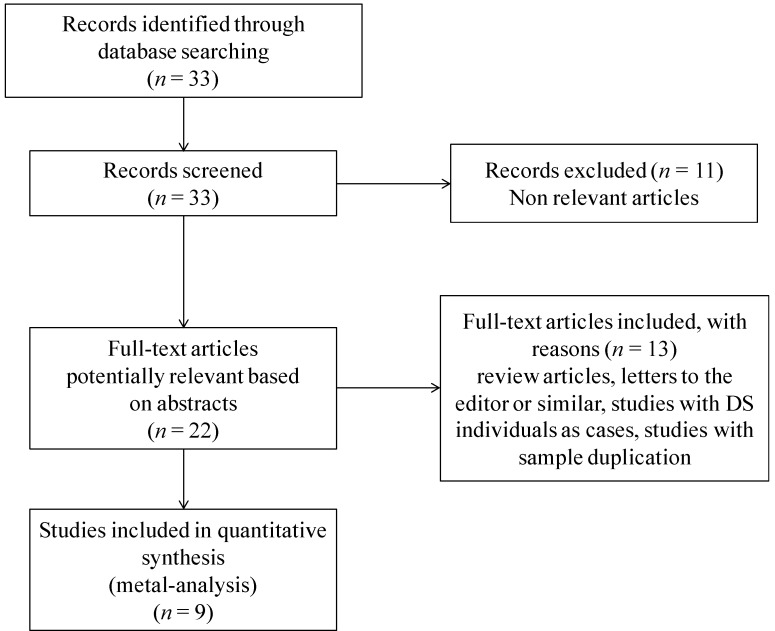
Flow chart of retrieved studies and studies excluded, with reasons specified.

The electronic PubMed and Scopus databases were searched up until March 2013 for the studies on *RFC-1* genetic polymorphisms as maternal risk factors for the birth of a child with DS, by means of the following terms: “Reduced folate carrier and Down syndrome”, “RFC and Down syndrome”, “RFC1 and Down syndrome”, “RFC-1 and Down syndrome”, and “SLC19A1 and Down syndrome”. To avoid a possible loss of any relevant article, an additional control was performed through the references cited in identified articles, and through the link “related articles” offered in the PubMed database. The literature review identified 33 titles that met the searching criteria. The abstracts of the retrieved studies were read to assess their appropriateness for inclusion in the meta-analysis. Figure 1 presents a flow chart of the retrieved studies and the studies excluded, with specifying reasons. Eleven studies have been excluded because of being not relevant, most of them dealing with the treatment of acute leukemia in children with DS, RFC-1 being responsible for the intracellular transport of methotrexate [33]. Of the 22 remaining potentially relevant articles, two were review articles, five studies were performed on DS individuals as cases, two studies were not genetic association studies, and two studies were letters to the editor discussing previously published data. After removal of those articles, 11 retrospective case-control studies, in all languages, evaluating the association of the *RFC-1* c.80A>G polymorphism and maternal risk for having a birth with DS, and showing tabular data, were recorded. The studies by the same author were controlled for a possible overlapping of included patients: three such articles were found [22,29,34], and only the more recent study with a higher number of cases was selected [29]. After removing studies with data overlapping, nine studies were found suitable for inclusion in this meta-analysis (Figure 1).

For each study the following data were extracted: author and year of publication, country, ethnicity and, when available, data on age at delivery (Table 1). Table 2 shows allele and genotype distributions for *RFC-1* c.80A>G in the selected studies. Deviations from Hardy-Weinberg equilibrium (HWE) in controls were evaluated by means of chi-square analysis (χ^2^), and only one study [27] showed deviations from HWE expectations, likely because of the very small sample size.

**Table 1:** Characteristics of the studies included in the meta-analysis.

Author and year [Ref.]	Country	Ethnicity	MDS/MC	Age at delievry
Chango *et al.* 2005 [25]	France	white Caucasians	119/94 ^a^	MDS: 33.8 ± 6 years MC: 29.5 ± 6 years
Coppedè *et al.* 2006 [19]	Italy	white Caucasians	69/93	MDS and MC: both aged <35 years
Scala *et al.* 2006 [20]	Italy	white Caucasians	94/263	MDS: 32.4 ± 6.3 years MC: 30 ± 5.6 years
Fintelman-Rodrigues *et al.* 2009 [26]	Brazil	Brazilian (mixed)	114/110	MDS and MC: both aged <35 years
Brandalize *et al.* 2010 [24]	Brazil	Brazilian (whites)	239/197	MDS: 121 ≥ 35 years ^b^ MC: 29 ≥ 35 years
Liao *et al.* 2010 [23]	China	Asians	60/68	not available: article in Chinese
Neagos *et al.* 2010 [27]	Romania	white Caucasians	26/46	MDS and MC: range 20–42 years
Zampieri *et al.* 2012 [29]	Brazil	Brazilian (mixed)	105/185	MDS: 54 ≤ 35 years ^b^ MC: 173 ≤ 35 years
Wang *et al.* 2013 [21]	China	Asians	104/184	MDS and MC: both aged <35 years

MDS: mothers of DS children; MC: control mothers; Age at delivery: expressed to show cases aging more or less than 35 years at delivery or, if not possible, as mean ± standard deviation or range; ^a^ Experience of abortion or miscarriage in some control mothers; ^b^ Significant prevalence of older mothers in the case group.

**Table 2:** Distribution of *RFC-1* 80A>G alleles and genotypes in Down Syndrome mothers and control mothers.

Author and year [Ref.]	MDS Alleles	MC Alleles	MDS Genotype	MC Genotype	HW	*p*-Value
Chango *et al.* 2005 [25]	Allele A: 114	Allele A: 84	AA:24/AG:6/GG:2	AA:16/AG:5/GG:2	HWE yes	*p* = 0.24
	Allele G: 124	Allele G: 104				
Coppedè *et al.* 2006 [19]	Allele A: 55	Allele A: 82	AA:13/AG:29/GG:2	AA:20/AG:42/GG:3	HWE yes	*p* = 0.42
	Allele G: 83	Allele G: 104				
Scala *et al.* 2006 [20]	Allele A: 95	Allele A: 317	AA:27/AG:4/GG:2	AA:102/AG:113/GG:4	HWE yes	*p* = 0.09
	Allele G: 93	Allele G: 209				
Fintelman-Rodrigues *et al.* 2009 [26]	Allele A: 114	Allele A: 113	AA:25/AG:64/GG:25	AA:29/AG:55/GG:2	HWE yes	*p* = 0.99
	Allele G: 114	Allele G: 107				
Brandalize *et al.* 2010 [24]	Allele A: 247	Allele A: 219	AA:73/AG:101/GG:65	AA:64/AG:91/GG:4	HWE yes	*p* = 0.36
	Allele G: 231	Allele G: 175				
Liao *et al.* 2010 [23]	Allele A: 70	Allele A: 64	AA:24/AG:22/GG:14	AA:12/AG:40/GG:16	HWE yes	*p* = 0.14
	Allele G: 50	Allele G: 72				
Neagos *et al.* 2010 [27]	Allele A: 18	Allele A: 40	AA:1/AG:16/GG:9	AA:5/AG:30/GG:11	**HWE no**	***p* = 0.02**
	Allele G: 34	Allele G: 52				
Zampieri *et al.* 2012 [29]	Allele A: 106	Allele A: 194	AA:29/AG:48/GG:28	AA:53/AG:88/GG:44	HWE yes	*p* = 0.53
	Allele G: 104	Allele G: 176				
Wang *et al.* 2013 [21]	Allele A: 135	Allele A: 271	AA:47/AG:41/GG:16	AA:100/AG:71/GG:13	HWE yes	*p* = 0.94
	Allele G: 73	Allele G: 97				

MDS: mothers of DS children; MC: control mothers; HWE: Hardy-Weinberg equilibrium.

### 2.2. Statistical Analysis

A meta-analysis of the selected studies (Table 1, Table 2) was performed to examine the association of the maternal *RFC-1* c.80A>G polymorphism with the risk of birth of a child with DS. Allele contrast, recessive and dominant model of the minor allele (*RFC-1* 80G) were evaluated. Pooled odds ratios (ORs) and 95% confidence intervals (CI) were obtained using the Mantel-Haenszel fixed effects model [35] and the DerSimonian-Laird random effects model [36]. The Chi-squared based *Q*-test and *I*^2^ were calculated to evaluate heterogeneity between studies [37,38], and a *p*-value > 0.10 for the *Q*-test was considered as indicating lack of heterogeneity between studies: in this case the pooled OR obtained using the fixed effects model was considered to be an appropriate estimate of the association. Heterogeneity in meta-analysis refers to the variation in study outcomes between studies, and the classical measure of heterogeneity is the *Q*-test. *Q* is distributed as a chi-square statistic with *k* (number of studies) minus 1 degree of freedom, and is usually included in each meta-analysis because it forms part of the DerSimonian-Laird random effects pooling method [36,37]. The *I*^2^ statistic describes the percentage of variation across studies that is due to heterogeneity rather than chance. Unlike *Q*, *I*^2^ does not inherently depend upon the number of studies considered, so that it describes the proportion of total variation in study estimates that is due to heterogeneity [37,38]. If there is no, or little, heterogeneity among studies, then *I*^2^ will be low and a fixed effects model is appropriate for the meta-analysis. Using a fixed effects model, all the studies under examination are considered to have been conducted under similar conditions with similar subjects, in other words, we assume that there is one true effect size, which is shared by all the included studies and all differences in observed effects are related to the random error inherent in each study. The global effect is therefore obtained as a weighted mean, with weight assigned to each study reflecting the within study variance. In the case of a significant heterogeneity among studies, the random effects model is considered to be more appropriate. Under the random effects model, the studies included in the meta-analysis are assumed to be a random sample of effect sizes that could have been observed, and the combined effect estimates the mean of these effects. The overall mean effect is obtained as a weighted mean, where weight assigned to each study is the inverse of study’s variance and, in this case, the variance includes both within and between study variance [39,40].

Sensitivity analyses were performed excluding the one study [27] with HWE deviations in controls and both the previous one and the study in which control mothers had experience of abortion or miscarriage [25].

## 3. Results

A total of nine studies were identified and considered in the meta-analysis (Table 1). HWE was verified in each study and the *p*-value, resulting from χ^2^ analysis, indicated departure from the equilibrium only in one study [27]. Overall, a total of 930 case-mothers and 1240 control mothers were used to examine the association between the *RFC-1* 80A>G polymorphism and maternal risk of birth of a child with DS, and results are shown in Table 3. The main finding of the study was a significant association under the recessive genetic model (GG *vs*. AG or AA), with an OR = 1.27 (95% CI: 1.04–1.57) if all the studies were evaluated, and an OR = 1.26 (95% CI: 1.02–1.55) after removal of the study with HWE deviations in controls. Similar results were obtained when removing both the one study with HWE deviations and the one in which control mothers had previous experience of miscarriage/abortion: OR = 1.33 (95% CI: 1.06–1.66). Since heterogeneity among studies was not significant, we used the fixed effects model to estimate the association; however, similar results were obtained also with the random effects model (Table 3). HWE deviations in control cohorts are frequently caused by genotyping errors or relatively low sample size [14]. Concerning the one study with HWE deviations [27], it was likely due to low sample size in that cohort (Table 1). However, since we could not exclude genotyping errors we performed the meta-analysis either including or excluding that paper. Similarly, as part of a sensitive analysis, we also decided to exclude the one study [25] that included control mothers who had experience of miscarriage/abortion [25], making that paper of questionable value for inclusion in the meta-analysis because of chromosome non-disjunction events are one of the leading causes of miscarriage/abortion [14]. Our observation of a still significant effect, even after removal of those studies that included questionable control cohorts, adds value to the association of the studied polymorphism with the maternal risk for having a birth with DS.

**Figure 2:**
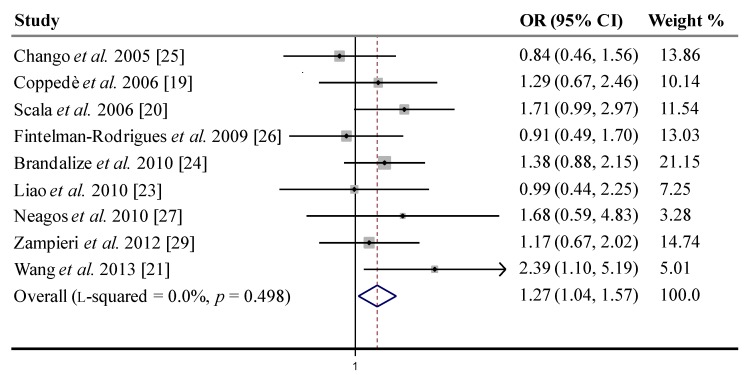
Fixed effects pooled odds ratio (OR) and 95% confidence intervals (CI) forest plot for the association between *RFC-1* 80A>G polymorphism and maternal risk for a DS birth in case-control studies, under the genetic recessive model, *i.e*., GG *vs*. (AG or AA). The OR estimate for each study is marked with a solid grey square. The size of the square represents the weight that the corresponding study exerts in the meta-analysis. The pooled OR is represented by a diamond. The CI of the pooled estimates are displayed as the horizontal margins of the diamond.

A forest plot with results of individual and summary OR estimates with 95% CI (fixed effects model) for *RFC-1* 80A>G recessive genetic model is shown in Figure 2. In addition, the fixed effect model for allele contrast (G *vs*. A) gave significant results in the whole set of studies, with an OR = 1.14 (95% CI: 1.01–1.30), but this association was not significant after removal of the study with HWE deviations in controls, and again significant when removing both this study and the one in which control mothers experienced previous miscarriages/abortions (Table 3). No significant associations were observed under the dominant genetic model (GG or AG *vs*. AA), using both fixed or random effects estimates (Table 3).

**Table 3:** Results of the meta-analysis.

Genetic Model	Studies	Fixed effects	*p*-value	Random effects	*p*-value	*Q*-statistics	*p*-value ^a^	*I*^2^
		OR (CI95%)		OR (CI 95%)				
G *versus* A	All	**1.14 **	**0.032**	1.14	0.121	12.56	0.128	2.0%
		**(1.01–1.30)**		(0.97–1.33)				
	HWE	1.14	0.047	1.12	0.187	12.12	0.097	2.0%
		(1.00–1.29)		(0.95–1.33)				
	HWE, no M ^b^	**1.17 **	**0.019**	1.16	0.105	10.17	0.118	2.0%
		**(1.02–1.34)**		(0.97–1.39)				
Dominant	All	1.12	0.243	1.09	0.526	13.74	0.089	7.0%
		(0.92–1.36)		(0.83–1.43)				
	HWE	1.11	0.279	1.07	0.612	12.95	0.073	7.0%
		(0.91–1.35)		(0.81–1.42)				
	HWE,	1.14	0.194	1.11	0.516	12.09	0.06	8.0%
	no M ^b^	(0.93–1.40)		(0.82–1.50)				
Recessive	All	1.27	0.020	1.27	0.020	7.37	0.5	0.0%
		(1.04–1.57)		(1.04–1.57)				
	HWE	1.26	0.030	1.26	0.031	7.09	0.42	0.0%
		(1.02–1.55)		(1.02–1.56)				
	HWE,	1.33	0.012	1.33	0.012	5.21	0.517	0.0%
	no M ^b^	(1.06–1.66)		(1.07–1.67)				

Genetic models: G *vs*. A: allele contrast; Dominant model: GG or AG *vs*. AA; Recessive: GG *vs*. AG or AA; ^a^ The *p-*value is referred to the *Q* statistcs to test for heterogeneity. ^b^ HWE, no M = only studies with no deviations from HWE in controls and with no experience of miscarriage/abortions in controls were included.

## 4. Discussion

Results of the present meta-analysis suggest a possible role for the *RFC-1* 80A>G polymorphism in modulating maternal risk for having a child with DS, and particularly an increased maternal risk for carriers of the homozygous *RFC-1* 80GG genotype. Those data are in agreement with several previous studies observing an increased maternal risk for trisomy 21 in individuals carrying the *RFC-1* 80GG genotype either alone [20,21] or combined with other polymorphic alleles of genes involved in folate metabolism [19,22,24]. Moreover, a functional role for the studied polymorphism has been suggested, with some authors observing association with reduced serum or red blood cell folate contents [18,27,28]. In addition, maternal *RFC-1* genetic polymorphisms known to be in linkage disequilibrium with the 80A>G one have been associated with an increased risk for congenital heart defects in the DS offspring [32], overall indicating a possible contribution of maternal variants in this gene in modulating both the risk for chromosome 21 malsegregation as well as the onset of DS-associated diseases in the offspring. Moreover, children with DS have a significantly higher risk of developing leukemia compared to non-DS children [33]. RFC-1 is responsible for the intracellular transport of the chemotherapeutic drug methotrexate, and cancerous cells of DS subjects with extra copies of chromosome 21 are at increased risk of methotrexate-associated toxicity due to an increased intracellular transport of the drug via RFC-1 [33].

The odds ratios and 95% confidence intervals obtained using both fixed and random effects models were very similar under the recessive genetic model (GG *vs*. AA or AG). By contrast, some differences were observed concerning the allele contrast (G *vs*. A) model. Those differences can be explained taking into account both *Q* and *I^2^* values and considering that under the fixed effects model, by definition, the overall estimates is more influenced by larger studies and information coming from small studies has a small weight, while in the random effects model, since each study is assumed to provide information about a different effect size, all these effect sizes are represented in the summary estimate [39,40]. As shown in Table 3, under the recessive genetic model, heterogeneity among studies was not significant, and *I*^2^ was equal to 0, indicating that 0% of variation across studies was due to heterogeneity rather than chance, and explaining why we obtained very similar results using either a fixed or a random effects model. Conversely, under the allele contrast model heterogeneity among studies was still low, but the *I*^2^ value revealed that 2% of the variation across studies was due to heterogeneity. In this case 95% confidence intervals were wider under the random effects model than under the fixed effects one, leading to different outcomes.

### Limitations

Albeit largely promising, present results deserve additional discussion and confirmation in subsequent well-designed studies. Among factors that must be taken into account when assessing the contribution of folate gene polymorphisms to the maternal risk for chromosome 21 malsegregation are maternal age and the use of folate supplements at periconception, as well as the type of meiotic error (*i.e*., occurred at maternal meiosis I or II) that caused chromosome 21 nondisjunction [14,41]. A brief look at Table 1 clearly shows the heterogeneity of the case-control studies, so far available, in terms of maternal age at delivery, with few studies performed only in women aging less than 35 years [19,21,26], others performed in matched case-control cohorts including both women aging more or less than 35 years at delivery [20,25,27], and even studies performed in unmatched case-control cohorts with a significant prevalence of aged MDS than aged control mothers [24,29]. Despite this, all those papers have been included in previous meta-analyses of the literature [15,16,17], such a heterogeneous group of available data renders almost impossible to assess the contribution of the maternal age effect in a meta-analysis. In addition, data from individual case-control studies are conflicting, including authors observing an increased effect of the *RFC-1* 80GG genotype with increasing maternal age [20], and others suggesting an increased maternal risk for a DS birth in young women carrying the *RFC-1* 80GG genotype [21]. Moreover, none of the studies listed in Table 1 shows tabular data stratified according to errors occurred at maternal meiosis I or II, and data on folate availability or supplements at periconception are scarce. Only future case-control studies designed to take into account all the above variables will help to further elucidate the contribution of folate gene polymorphisms to the maternal risk for trisomy 21 in the offspring [14].

Another factor deserving further investigation is the contribution of ethnicity. Given the paucity of available comparable case-control studies for each ethnic group included in the present meta-analysis (Table 1), we did not perform data stratification according to ethnic groups. Particularly, only two studies were available in Asians [21,23], and the three studies performed in Brazil were not comparable in terms of ethnic composition, since two of them were performed in mixed Brazilian populations [26,29], whilst the third one only included white Brazilians [24]. Moreover, two [25,27] out of the four [19,20,25,27] studies performed in European Caucasians have weaknesses, with the first showing deviations from HWE equilibrium in controls [27], and the latter including women experiencing abortions or miscarriages in controls [25].

## 5. Conclusions

The present meta-analysis of the literature reveals a significant increased maternal risk for having a birth with DS in carriers of the *RFC-1* 80GG genotype, suggesting that the studied polymorphism deserves further consideration. Additional case-control studies are required to clarify the joint effect of dietary factors, maternal age at conception, and the type of meiotic error, as well as the contribution of ethnic and geographic factors.
